# Introduction of Stilbene Derivatives and Cinnamate Ester Derivatives at the ω-End Groups of Poly(Methyl Methacrylate) Prepared via RAFT Polymerization

**DOI:** 10.3390/polym12112449

**Published:** 2020-10-23

**Authors:** Martyn Dobinson, Philip Hodge, Trevor Wear

**Affiliations:** 1 Department of Chemistry, University of Manchester, Oxford Road, Manchester M13 9PL, UK; Martyn.dobinson@saffery.com; 2 Kodak European Research, 332 Cambridge Science Park, Milton Road, Cambridge CB4 0WN, UK; trevor.wear@gmail.com

**Keywords:** capping, “living” poly(methyl methacrylate), “living” polystyrene, stilbene derivatives, cinnamate esters

## Abstract

The capping of “living” poly(methyl methacrylate) (PMMA) and “living” polystyrene (PS), both prepared by the RAFT technique, with various olefins was screened using ^19^F-NMR spectroscopy. The capping of “living” PMMA with a labeled stilbene was as high as 63% and with certain cinnamate esters was essentially quantitative, but the capping of “living” polystyrene with all the olefins investigated was generally poor.

## 1. Introduction

There is a longstanding interest in the synthesis of vinyl polymers with particular end groups. Appropriate end group functionalities could possibly be used, for example, (i) to bind the polymer via the end groups to a particular surface [1]; (ii) to provide recognition sites at the polymer chain ends; (iii) to fluorescently label the end groups of the polymer [2]; or (iv) to synthesize polymers with novel architectures [3].

In the 1980s and 90s, Bevington’s research group studied the free radical polymerization of methyl methacrylate and styrene in the presence of small amounts of stilbenes, or related olefins. These polymerizations were monitored by using starting materials incorporating ^13^C–, ^14^C– or ^19^F–labels [4,5,6,7,8,9,10,11]. They found that when benzoyl peroxide (**1**) is used as the initiator, the benzoyloxy radicals, produced from the initial thermal decomposition of (**1**), react much more rapidly with the stilbene, or related olefins, than with the methacrylate or styrene monomer—so rapidly, in fact, that relatively few of the benzoyloxy radicals have a chance to decarboxylate to produce phenyl radicals. As a consequence, many of the benzoyloxy initiator fragments at the α-end of the growing polymer chain have an adjacent stilbene residue.


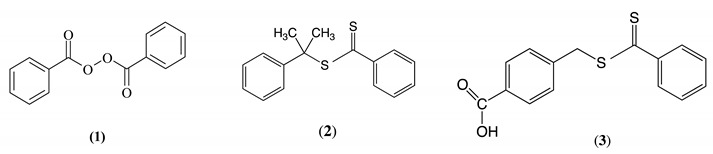


**Scheme 1:**
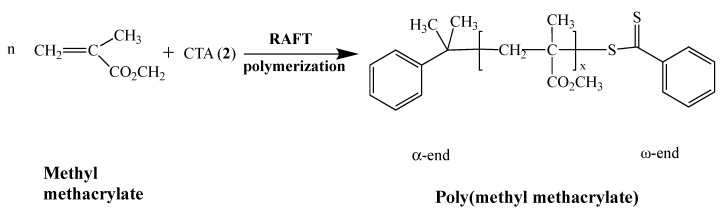
Example of a RAFT polymerization in the presence of CTA (**2**). The polymerization may also be initiated by an added organic peroxide. The latter may allow the polymerization to be initiated at a lower temperature.

Poly(methyl methacrylate) (PMMA) and polystyrene (PS) with functional end groups can also be obtained via reversible addition–fragmentation transfer (RAFT) polymerization [12,13,14,15,16,17]. RAFT is a versatile radical polymerization method that in many cases enables the preparation of polymers with predetermined molecular weights and narrow molecular weight distributions. Control is achieved by the use of a chain transfer agent (CTA). Often, the chosen CTA is cumyl dithiobenzoate (**2**) and this leads to cumyl groups as the α-end groups and dithioester groups as the ω-end groups: see Scheme 1. The latter react very rapidly with the small number of free radical chain ends. The result is the rapid and reversible transfer of the dithioester groups between the many chain ends. This means that all the polymer chains have an equal chance of reacting with the monomer and the final polymer has a narrow molecular weight distribution and a predictable molecular weight.

Using RAFT polymerization, there are three main approaches for the synthesis of polymers with functional end groups [2,16,17]. First, to functionalize the α-end, an appropriately functionalized CTA is used. CTA (**3**) is an example [16]. Second, a thiol can be obtained at the α-end by reacting the terminal dithioester with, for example, *n*-heptylamine [17,18,19]. Subsequently, if desired, the thiol can then undergo a thiol-ene addition with an olefin [20]. Third, allow the “living polymer” ω-end groups to react with an olefin. If the olefin simply adds on and then propagates, as happens with, for example, styrene or methyl methacrylate, a block copolymer is formed [12,13]. However, if the olefin adds on but does not propagate, just one olefin unit is added, i.e., the chain is capped. An example is the capping of “living” PMMA with maleic anhydride [2,3]. This is most likely to be the case with non-homopolymerizable olefins. If the ω-end is still “living”, a second polymer chain may be grown. A successful example of this is the addition of maleic anhydride to “living” PS followed by elaboration to give a three-armed star polymer where all three arms are a different polymer [3].

The aim of the present project was to investigate the capping of “living” PMMA and PS, prepared by RAFT polymerization, with various olefins. In particular, the aim was to develop a method for rapidly screening the capping reactions using ^19^F NMR spectroscopy. Initially, it was hoped that it would prove possible to add single stilbene groups efficiently to the ω-ends of the chains.

## 2. Materials and Methods

### 2.1. Materials

All the materials used were purchased from Aldrich, Lancaster Synthesis or Fluorochem and, unless otherwise stated, were used without further purification. Commercial benzoyl peroxide (**1**) was purified by recrystallization from methanol before use and stored at 2–3 °C in a refrigerator. The styrene monomer (b.pt. 142–146 °C at 760 mm of Hg) as purchased was stabilized with 10–15 ppm 4-t-butylcatechol (b.pt. 285 °C at 760 mm of Hg) and was purified before use by distillation under reduced pressure. The methyl methacrylate monomer (b.pt. 100 °C at 760 mm of Hg) as purchased was inhibited with 10–100 ppm 4-methoxyphenol (b.pt. 240 °C) and was purified by distillation. All reactions were carried out under atmospheric conditions.

### 2.2. Conventional Free Radical Polymerization

The peroxide initiator (**1**) or (**4**) (0.5 mol%, 2.4 × 10^−4^ mol) was dissolved in the relevant monomer (0.048 mol) and transferred into the polymerization vessel. Toluene solvent (6.87 mL) was added. The contents of the vessel were then degassed by at least 3 successive freeze–evacuate–thaw cycles on a vacuum line fitted with an Edwards High Vacuum pump (Edwards Vacuum Company, Burgess Hill, UK), using liquid nitrogen for freezing. The vessel was then sealed and heated at 85 °C under vacuum for 67 h. The contents of the vessel were cooled before being added dropwise to a large excess of a suitable non-solvent for the polymer, in which monomer and residual initiator were soluble. The polymeric product precipitated and was removed by suction filtration, redissolved in a minimum quantity of toluene and reprecipitated a second and then a third time, using fresh large excesses of the non-solvent. The polymer was then dried under vacuum and characterized.

### 2.3. Standard Method for RAFT Free Radical Polymerization

The relevant peroxide initiator, (**1**) or (**4**) (0.5 mol%, 2.4 × 10^−4^ mol), and the relevant RAFT agent, (**2**) or (**5**) (1 mol%, 4.8 × 10^−4^ mol), were dissolved in the relevant monomer (0.048 mol) and transferred into the polymerization vessel. Toluene solvent (6.87 mL) was added. The contents of the vessel were degassed by at least 3 successive freeze–evacuate–thaw cycles on a vacuum line fitted with an Edwards High Vacuum pump, using liquid nitrogen for freezing. The vessel was sealed and heated at 85 °C under vacuum for 67 h. The reaction mixture was cooled to 20 °C and added dropwise into non-solvent. The polymeric product precipitated out and was collected. It was reprecipitated a second and then a third time, using fresh large excesses of the non-solvent. The polymer was then dried and characterized.

### 2.4. Chain Extension of the “Living” Polymers with the Non-Homopolymerizable Monomers: “Capping”

The relevant “living” polymer (200 mg) and the relevant chain extension agent (10× molar excess, relative to the number of moles of polymer chains in the respective sample) were dissolved in *m*-xylene (3 mL) and then transferred into the polymerization vessel. The contents of the vessel were degassed by at least 3 successive freeze–evacuate–thaw cycles on a vacuum line fitted with an Edwards High Vacuum pump, using liquid nitrogen for freezing. The vessel was then sealed and heated at 100 °C under vacuum for 67 h. The contents of the vessel were cooled before being added dropwise to a large excess of a suitable non-solvent for the polymer, in which residual chain extension agent was soluble, in order to precipitate and purify the chain extended polymer. The polymeric product was removed by suction filtration, redissolved in a minimum quantity of toluene and reprecipitated a second and then a third time, using fresh large excesses of the non-solvent. The polymer was then dried and characterized.

### 2.5. Modified Method for Capping the “Living” Polymers

The relevant “living” polymer (200 mg) and the relevant chain extension agent (2× molar excess, relative to the number of moles of polymer chains in the respective sample) were dissolved in *m*-xylene (3 mL) and the solution transferred into the polymerization vessel. The polymerization was continued as above.

### 2.6. Size Exclusion Chromatography (SEC)

All SEC analyses were performed using the following system, which was assembled in house. Column system: PL Gel 30 cm 10 μ − 2× mixed B + 1× 500 Å (Polymer Laboratories, Church Stretton, UK); temperature: ambient; Detector: Gilson 132 Differential Refractometer (Gilson Inc, Middleton, USA); temperature: ambient; sensitivity: 0.3 (10^−4^ RIU/FS); Pump: Knauer 64; eluent flow rate: 1 mL/min; solvent: THF; injection volume: 100 μL; concentration: 0.2% or 2 mg/mL. Unless otherwise stated, all molecular masses were calibrated against narrow polydispersity polystyrene standards of the following molecular masses and are quoted in units of Daltons (atomic mass units, Da): 220, 1010, 2100, 10,000, 17,500, 51,000, 110,000, 200,000, 1,800,000. The internal standard used was *n*-dodecane.

### 2.7. ^19^F-Nuclear Magnetic Resonance Spectroscopy

The ^19^F-NMR spectra were recorded on a Varian Inova-400 (400 MHz) Spectrometer with dichloromethane as solvent. All chemical shifts are quoted on the δ-scale in units of parts per million (ppm), relative to hexafluorobenzene and using residual protonated solvent as the internal standard.

### 2.8. MALDI-ToF Mass Spectrometry (MALDI-ToF MS)

The matrix-assisted laser desorption ionization—time of flight mass spectra (MALDI-ToF MS) were recorded on a Micromass ToF Spec 2E Spectrometer. The dried drop method of sample preparation was used. Dithranol and 2,5-dihydroxybenzoic acid (DHB) were the matrices employed. Sodium trifluoroacetate and silver trifluoroacetate were the dopant salts employed. Sample, matrix and salt solutions were made up in a common solvent, generally either chloroform or THF, at concentrations of 10 mg/mL. A droplet of the salt solution, typically 1–2 μL, was spotted into a well on a 100-well stainless steel Micromass ISS3 MALDI-ToF mass spectrometry plate. The solvent was allowed to evaporate. The matrix and sample solutions were then mixed in a ratio of 10:1, matrix: sample. A droplet of this solution, typically 1–2 μL, was deposited in the well on the MALDI-ToF mass spectrometry plate. The solvent was allowed to evaporate. All MALDI-ToF mass spectra were calibrated against the sodium salt of a lauric acid—caprolactone polymer (repeat unit of 114 Da) run in the DHB matrix.

The instrument was operated using the following parameters. Instrument mode: reflectron; operating voltage: 20 kV; polarity: positive; source voltage: 20,000 V; extraction voltage: 19,950 V; focus voltage: 17,000 V; reflectron voltage: 26,000 V; detector voltage: 1706 V; laser: N_2_ UV laser at a wavelength of 337 nm with a pulse width of 4 ns; suppression mass: 500 Da; pulse time: 39 ns; pulse voltage: 2900 V; sampling rate: 500 MHz; sensitivity: 500 mV; shots: 5; mass range: 6000 Da; laser coarse: 50% energy; laser fine: 80; laser shots per second: 5; update peak display every 5 shots. All spectra were processed in the same way.

The illustrated spectra are the combination of at least 15 individual spectra. They were smoothed using one Savitzky Golay smooth ±1 channel, and the background subtraction was performed using a polynomial of order 8 with 10% below the curve and a 0.01 tolerance.

## 3. Results

### 3.1. Preliminary ^19^F NMR Spectroscopy Studies

**Chart 1:**
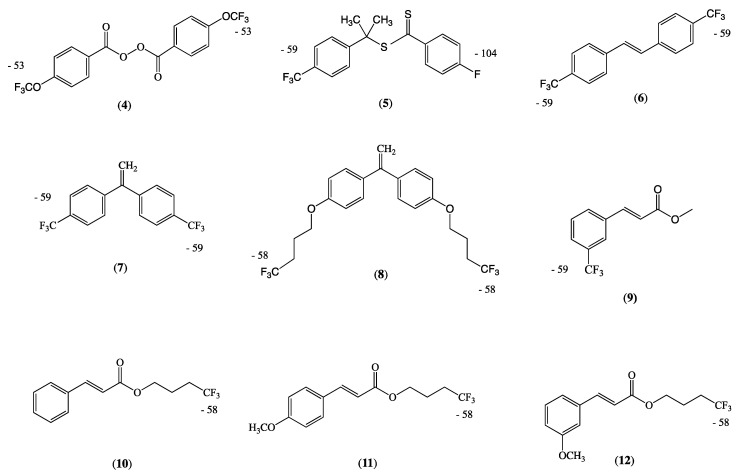
Fluorine-containing compounds (**4**)–(**12**). The spectra were measured for solutions in dichloromethane at 25 °C. The formulae are labeled with the chemical shifts (δ ppm) of the fluoro residues relative to fluorobenzene, which is taken as 0 ppm. Under these conditions, all the shift values are negative.

For the present study, there was a need for an ^19^F-labeled benzoyl peroxide, a labeled cumyl dithiobenzoate and the various labeled olefins whose capping ability was to be investigated. This prompted the synthesis of compounds (**4**)–(**12**): see Chart 1. These syntheses were achieved using similar procedures to those used for the synthesis of the corresponding compounds that lack the fluorine-containing groups: see Appendix A for details of the syntheses. In several cases, 4,4,4-trifluorobutyl labels were used because the fluoro groups were well removed from the olefinic bonds and hence unlikely to have any significant effect on their reactivity.

Also shown in the Chart are the ^19^F-chemical shifts of the various fluorinated groups. It will be noted that the shifts fall into three well-resolved regions: δ -53 ppm for the CF_3_O– residues of the labeled peroxide (**4**); δ -104 ppm for the “living” dithiobenzoate fluoro group; near δ -58 ppm for the initiator part of CTA (**5**) and for all the potential capping olefins.

**Figure 1:**
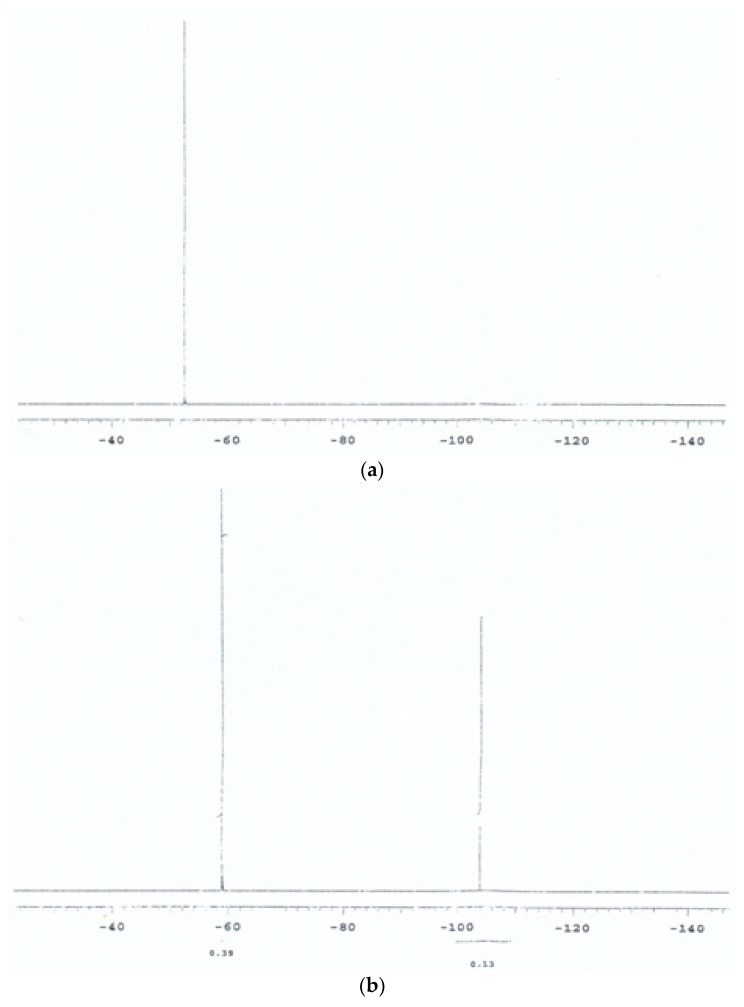
^19^F-NMR spectra of compounds (**a**) (**4**) and (**b**) (**5**) measured for solutions in dichloromethane at 25 °C. Chemical shifts (δ ppm) of the fluoro residues are given relative to hexafluorobenzene, which is taken as 0 ppm. Note that all the shift values are negative.

^19^F NMR spectra for (**4**) and (**5**) are shown in Figure 1. Note that the signals derived from the peroxide initiator at δ -53 ppm are well resolved from the other initiator fragment at δ -59 ppm that derives from CTA (**5**). This is an important point in the analysis of the many of the spectra discussed below as it allows the fraction of the signal near δ -58 ppm that is due to the initiator and the fraction that is due to the capping olefin to be determined.

### 3.2. Syntheses of “Living” PMMAs (13) and (15) and “Living” PS (14)

Initially, PMMA and PS homopolymers were prepared both by standard free radical initiated polymerizations and by the RAFT polymerization technique but using compounds (**4**) and (**5**) in place of benzoyl peroxide (**1**) and the common unlabeled CTA (**2**), respectively. The results are summarized in Table 1. The polymerizations summarized in entries 1 and 2 are non-RAFT polymerizations simply using the labeled benzoyl peroxide (**4**) as the initiator. The results suggest that the polymerizations occur similarly to polymerizations initiated by benzoyl peroxide (**1**). The polymerizations summarized in entries 3 and 4 are RAFT polymerizations using CTA (**5**) plus a relatively small amount of the peroxide (**4**) to allow the polymerizations to be initiated at 85 °C. In the absence of the peroxide, the polymerization temperature would typically need to be ca. 110 °C [12]. The reaction conditions chosen for the RAFT polymerization are very similar to those used in our previous work on capping with maleic anhydride [2]. The unusual choice of 67 h for the reaction times arises from starting a reaction late one afternoon, then isolating the product in the morning two days later. The molecular weights of the products are low by choice to allow MALDI-MS. The results suggest that the polymerizations occur similarly to polymerizations carried out using benzoyl peroxide (**1**) and CTA (**2**).

The polymerizations were carried out in toluene at a monomer concentration of 4.0 mol dm^−3^, at a reaction temperature of 85°C, under vacuum and for a period of 67 h after being degassed. The PMMA (**13**) and PS (**14**) were recovered by precipitation, purified by reprecipitation, dried and then characterized by size exclusion chromatography (SEC) and by ^19^F-NMR spectroscopy. The polymerization summarized in entry 5 was carried out similarly but so as to produce a higher molecular weight PMMA (**15**). The yields of the polymers (**13**)–(**15**) were relatively low due to the much slower rate of polymerization under RAFT conditions. By SEC, the dispersity for the “living” PMMAs (**13**) to (**15**) were 1.13, 1.11 and 1.22, respectively, and the percentages of “living” chains were 92%, 93% and 73%. The M_n_ values correspond to degrees of polymerization of 40, 32 and 496, respectively.

**Figure 2:**
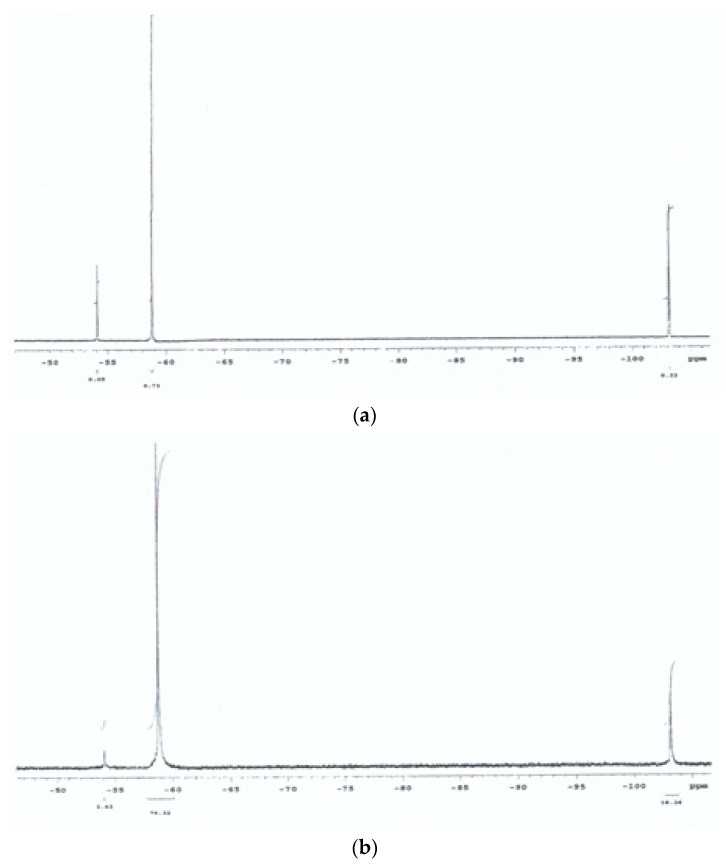
^19^F-NMR spectra of (**a**) polymer (**13**) and (**b**) polymer (**16**) measured for solutions in dichloromethane at 25 °C. Chemical shifts (δ ppm) of the fluoro residues are given relative to hexafluorobenzene, which is taken as 0 ppm. Note that all the shift values, as elsewhere, are negative.

^19^F-NMR spectra of polymers (**13**), (**14**) and (**15**) were measured. Consider, as an example, the spectrum of polymer (**13**); see Figure 2a. There are two important points. First, the spectrum shows signals due to the α-terminal groups deriving from peroxide (4), at δ -53 ppm, and the other initiator fragment that derives from the CTA (**5**) at δ -59 ppm. The sum of these two signals is proportional to the number of polymer chains present. The intensity of the 4-fluorodithiobenzoate signal at the ω-terminal (after due allowance is made for the number of fluorine atoms present in the respective groups) relative to the sum of the signals due to the α-terminal groups indicates the percentage of polymer chains that are “living”. Second, since, in subsequent capping experiments, the reaction conditions are not such as to affect the proportion of these α-end groups, the ratio of the two α-end signals allows the extent to which the signal near δ -59 ppm is due to CTA (**5**) and due to the capping agent under investigation.

### 3.3. Capping Reactions

The first potential capping agent studied was bis-4,4′-trifluoromethylstilbene (**6**). “Living” polymers (**13**) and (**14**) (200 mg of each) were separately treated with a ten-fold molar excess (relative to the number of moles of polymer chains in the respective sample) of the fluorine-labeled stilbene (**6**) by heating the polymers at 100 °C in *m*-xylene solution for 67 h, in sealed vessels, after the reaction solutions had been degassed by three successive freeze–evacuate–thaw cycles on a high vacuum line. The polymers were then recovered by precipitation, purified by reprecipitation, dried under vacuum, and the ^19^F-NMR spectra recorded. The results obtained with PMMA (**13**) are shown in Figure 2.

The cumyl initiating fragment of the CTA (**5**) was labeled with a trifluoromethyl group, as was the stilbene (**6**); hence, these two signals occurred in the same region of the ^19^F-NMR spectrum. Calculation from the integrals showed that 63% of the PMMA (**13**) chains were capped successfully and 100% of the chains were still “living”. With the PS (**14**) chains, a similar calculation showed that only 23% were capped by a stilbene unit. The results are summarized in Table 2.

The capping results with stilbene (**6**) are disappointing and prompted the study of derivatives of other types of non-polymerizable, or poorly polymerizable olefins, namely 1,1-diphenylethylenes (**7**) and (**8**) and cinnamate esters (**9**) to (**12**). These results are also summarized in Table 2.

Several points are evident.

(1) Capping of PMMA (**13**) was always much more successful than the capping of PS (**14**). Accordingly, all subsequent capping studies focused just on PMMAs.

(2) In the capping of PMMA (**13**), electron-donating substituents on the aromatic ring of the olefin increased the capping yield, whilst electron-withdrawing substituents decreased it.

(3) Capping with 1,1-diphenylethylene derivatives (**7**) and (**8**) was at best only moderately successful. The products of these capping experiments are interesting in that 1,1-diphenylethylene has been used with moderate success as a CTA [21]. Thus, these products can be seen as another type of “living” polymer.

(4) Cappings with cinnamate ester derivatives were the most successful capping experiments; in some cases, such as polymers (**17**) and (**18**), the yields were essentially 100%. 

**Figure 3:**
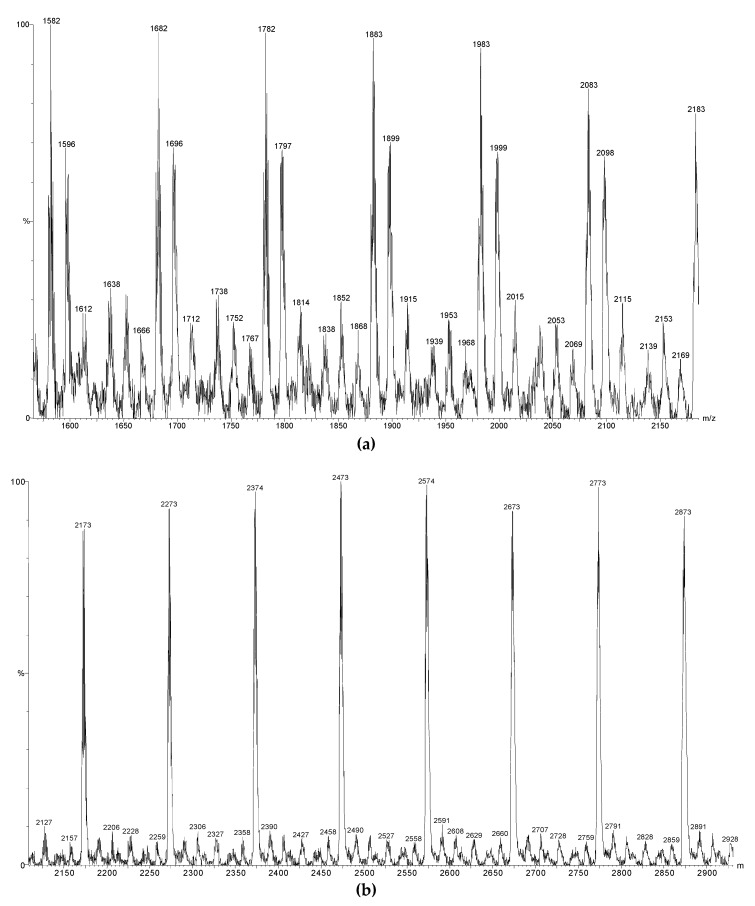

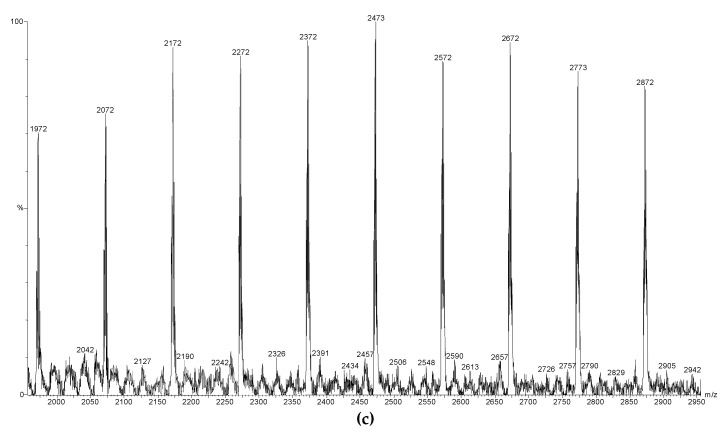
MALDI ToF mass spectra of (**a**) polymers (**13**), (**b**) polymer (**17**) and (**c**) polymer (**18**).

To obtain further evidence for the nature of the end groups in the more successful cases, the polymers obtained, PMMAs (**17**) and (**18**), were subjected to end group analysis using MALDI ToF mass spectrometry: see Figure 3.

Considering first the MALDI-ToF mass spectrum of “living” capped PMMA (**13**) in Figure 3a, the methyl methacrylate repeat unit mass of 100 Da is clearly evident as the difference between adjacent peaks in the major series. Taking the mass peak at m/z 1983 as an example, this peak corresponds to a polymer with a degree of polymerization (DP) of 16. Subtracting the mass of 16 repeat units from the total polymer mass yields a residual mass due a m/z to end groups of 381 Da. This mass corresponds to that of a trifluoromethylcumyl end group (187 Da), arising from the CTA (**5**), a fluorodithiobenzoate end group (171 Da), also arising from (**5**), and an attached sodium ion (23 Da), required for ionization. The series of weaker peaks at around 16 higher than the major series may be assigned to that of the potassium ion (39 Da) adduct of the polymer. There may also be a contribution in this region from the sodium ion (23 Da) adduct of the corresponding polymer chain with trifluoromethoxybenzoyl (205 Da) and fluorodithiobenzoate (171 Da) end groups, where the trifluoromethoxybenzoyl end group has arisen from the peroxide initiator (**4**). Thus, the MALDI ToF mass spectrum shown in Figure 3a is consistent with that of “living” PMMA (**13**).

Considering next the MALDI-ToF mass spectrum of PMMA (**17**) in Figure 3b, the repeat unit mass of 100 Da is once again clearly visible as the mass difference between adjacent peaks in the major series of peaks. Taking the peak occurring at a m/z 2273 as an example, this peak corresponds to the polymer with a DP of 16. Subtracting the mass of 16 repeat units from the total polymer mass yields a residual mass for end groups of 671 Da. This corresponds to that of a trifluoromethylcumyl end group (187 Da) from CTA (**5**), a fluorodithiobenzoate end group (171 Da), also arising from CTA (**5**), an attached sodium ion (23 Da), required for ionization, and a cinnamate (**9**) residue of 288 Da. The potassium ion adduct (39 Da) of the corresponding polymer can be seen as a very weak intensity series at a m/z around 16 Da higher than that of the major oligomeric series. There are no major peaks consistent with unreacted polymer (**13**) at m/z 1983 or multiples of 100 higher, or polymer (**13**) minus the fluorodithiobenzoate group at m/z 1812, or the capped product which has lost the fluorodithiobenzoate group, i.e., is no longer “living”, at m/z 2102. Thus, the mass spectrum is fully consistent with polymer (**13**), which has been capped with cinnamate ester (**11**) and is still “living”.

Considering next the MALDI-ToF mass spectrum of PMMA (**18**) in Figure 3c, the methyl methacrylate repeat unit mass of 100 Da is once again clearly evident as the mass difference between adjacent peaks in the major series of peaks. Taking the peak occurring at a m/z 2272 as an example, this peak corresponds to the polymer with a DP of 16. Subtracting the mass of 16 repeat units from the total polymer mass yields a residual mass for end groups of 670 Da. This mass corresponds to that of a trifluoromethylcumyl end group (187 Da) arising from CTA (**5**), a fluorodithiobenzoate end group (171 Da), arising from (**5**), an attached sodium ion (23 Da), required for ionization and a residue from compound (**12**) of 288 Da. The potassium ion adduct (39 Da) of the corresponding polymer may also be observed as a weak intensity series at a m/z of around 16 higher than that of the major oligomeric series. There are no major peaks consistent with unreacted polymer (**13**) at m/z 1983 or multiples of 100 higher, or polymer (**13**) minus the fluorodithiobenzoate group at m/z 1812, or the capped product which has lost the fluorodithiobenzoate group, i.e., is no longer “living”, at m/z 2102. Thus, the mass spectrum is fully consistent with polymer (**13**), which has been capped with cinnamate ester (**12**) and is still “living”.

The possibility of reducing the 10-fold use of capping agent required was investigated briefly, but with no major success. The results are summarized in footnotes (c) and (d) in Table 2. Another variable investigated, see footnote (e), was the temperature used in the capping reaction. To raise this to 130^o^C, the reactions were carried out in m-xylene under nitrogen for 67 h. When PMMA (**13**) was treated with capping agent (**11**) and with (**12**), the percentage capped was still quantitative within experimental error but all the polymer chains ceased to be “living”. MALDI ToF mass spectrometry analysis suggested that the capped chains were terminated with hydrogen atoms. When the higher molecular weight PMMA, PMMA (**15**), was similarly treated with compound (**11**) and with compound (**12**), all the chains were capped successfully but again the “living” character was lost.

## 4. Conclusions

Attempts were made to cap “living” PMMA (**13**) and “living” PS (**14**), both prepared by the RAFT technique, by treating them with a 10-fold excess of various non-polymerizable (that is, non-polymerizable by radical polymerization) olefins in *m*-xylene at 100 °C for 67 h. The initiator (**4**), RAFT CTA (**5**), and the various capping agents were all labeled with ^19^F-substituents so that the reactions could be screened by ^19^F NMR spectroscopy. The results are summarized in Table 2.

The “living” PMMA (**13**) was capped in 63% yield by 1,4-bistrifluoromethyl-*E*-stilbene (**6**). Judging by the effect of aromatic substituents on the yields in other capping experiments, it is likely that a stilbene with a 4-alkoxy substituent on each aromatic ring would be capped in significantly higher than 63% yield.

The “living” PMMA (**13**) was capped essentially quantitatively with 4-methoxy- and 3-methoxy-cinnamate esters (**11**) and (12), but attempted cappings with 1,1-diphenylethylenes (**7**) and (**8**) gave poor results. All attempts to cap the “living” PS (**14**) gave poor results. The higher molecular weight “living” PMMA (**15**) was also capped successfully at 130 °C but all the “living” character was lost.

Future work will involve optimizing the capping reactions, preparing the 4- and 3-cinnamate esters of various hydrophilic compounds, e.g., sugars or poly(ethylene glycol)s, and attaching them to the ω-ends of PMMAs synthesized using RAFT.

## Figures and Tables

**Table 1:** Conditions and results for various homopolymerizations of methyl methacrylate (MMA) and styrene (S) ^a^.

Entry	Monomer	Initiator	Percentage of Initiator(s) ^b^	Polymer Yield	Polymer Designation	M_n_^c^	M_w_^c^	D ^c^	Percentage “Living” ^d^
1	MMA	(**4**)	0.5	65	----	75.0	159.2	2.12	--
2	S	(**4**)	0.5	59	----	26.2	50.5	1.92	--
3	MMA	(**4**) + (**5**)	0.5 + 1.0 ^e^	14	(**13**)	3.95	4.45	1.13	92
4	S	(**4**) + (**5**)	0.5 + 1.0 ^e^	25	(**14**)	3.30	3.65	1.11	93
5	MMA	(**4**) + (**5**)	0.033 + 0.0667 ^e^	27	(**15**)	49.6	60.2	1.21	75

^a^ All polymerizations were carried out in toluene for 67 h at 85 °C: see Materials and Methods for further details. Polymerizations described in entries 1 and 2 were traditional free radical initiated polymerizations but using the novel initiator (**4**) instead of benzoyl peroxide (**1**). Polymerizations described in entries 3, 4 and 5 are RAFT polymerizations using novel CTA (**5**) and, to allow polymerization at 85 °C, added peroxide (**4**) as indicated. ^b^ Mole%. ^c^ ×10^−3^ g/mole. By SEC relative to polystyrene standards. See Materials and Methods for full details of the instrument. D = dispersity. ^d^ By ^19^F-NMR spectroscopy. ^e^ RAFT polymerization using peroxide (**4**) and CTA (**5**) in the mole ratio shown. See Materials and Methods for further details.

**Table 2:** Conditions and results for various capping experiments with polymers (**13**) and (**14**).^a^

Entry	Capping Agent	PMMA (13)			PS (14)	
		Capped ^b^	Living ^b,c^	Designation	Capped ^b^	Living ^b,c^
1	(**6**)	63%	100%	(**16**)	23%	n.d.
2	(**7**)	29%	n.d.	---	6%	n.d.
3	(**8**)	39%	n.d.	---	6%	n.d.
4	(**9**)	50%	n.d.	---	15%	n.d.
5	(**10**)	78%	n.d.	---	12%	n.d.
6	(**11**)	102% ^d^	94%	(**17**)	15%	n.d.
7	(**12**)	100% ^e^	85%	(**18**)	8%	n.d.
8	(**11**) ^f^	100%	0 ^g^	---	---	---

^a^ Unless indicated otherwise, the polymer in *m*-xylene at 100 °C was treated for 67 h with a 10-fold excess of the capping agent relative to the number of moles of polymer chains in the sample. ^b^ By 19F NMR spectroscopy. ^c^ n.d. = not determined. However, many of these products were white or very pale pink, indicating that all or much of the dithioester group was lost. ^d^ When conditions were as in (a) but only a 2-fold excess of capping agent was used, the capping yield fell to 77%. ^e^ When conditions were as in (a) but only a 2-fold excess of capping agent was used, the capping yield fell to 83%. ^f^ The polymer in *m*-xylene at 130 °C was treated with a 2-fold excess of capping agent for 67 h. ^g^ By MALDI ToF MS analysis, the end group was then −H.

## Appendix A

The Appendix gives experimental details for the synthesis of fluoro compounds (**4**)–(**12**): see the Chart in main paper. The General Experimental Procedures are additional to those given in the main paper, and the reference numbers follow on from those in the main paper.

### Appendix A.1. General Experimental Procedures

Unless otherwise indicated, all organic products in solution were dried using anhydrous magnesium sulphate. The solvent was removed under reduced pressure on a rotary evaporator. The last traces of solvent were removed by drying the compounds in a vacuum oven at 45 °C overnight. Unless otherwise stated, all column chromatography was performed using Merck, grade 60, 70–230 mesh, 60 Å silica gel purchased from Aldrich. All melting and boiling points were recorded using a Gallenkamp Hot Stage Melting Point Apparatus and the quoted values, in units on the Celsius scale (°C), are the average of three measurements.

*Infrared (IR) Spectroscopy:* The IR absorption spectra were recorded on an ATI Mattson Genesis Fourier Transform Infrared (FTIR) Spectrometer. Liquid samples were run as liquid films sandwiched between two sodium chloride plates. Solid samples were run as evaporated films from dichloromethane solution onto a sodium chloride plate. Absorption bands (ν_max_) are reported in wavenumbers (cm-1).

*NMR Spectroscopy*: The ^1^H-NMR spectra were recorded on a Varian Inova-300 (300 MHz) Spectrometer (Varian Inc, Palo Alto, California, USA) for sample solutions in CDCl_3_, unless otherwise stated. All chemical shifts are quoted on the δ-scale in units of parts per million (ppm), relative to tetramethylsilane (TMS) and using residual protonated solvent as the internal standard. The quoted splitting patterns are abbreviated to singlet (s), doublet (d), triplet (t), quartet (q), multiplet (m) and broad (b). The ^19^F-NMR spectra were recorded on a Varian Inova-400 (400 MHz) Spectrometer with CD_2_Cl_2_ as the solvent. All chemical shifts are quoted on the δ-scale in units of parts per million (ppm), relative to hexafluorobenzene and using residual protonated solvent as the internal standard.

*Elemental Analysis (EA)*: Elemental analyses were carried out in the Microanalysis Laboratory, Department of Chemistry, University of Manchester.

*Thin Layer Chromatography (TLC)*: This was carried out using Polygram SIL G/UV254 0.2 mm thick silica gel plates with fluorescent indicator UV254.

### Appendix A.2. Syntheses of Fluorocompounds *(**4**)*–*(**12**)*

#### Appendix A.2.1. Synthesis of Bis-(4-trifluoromethoxybenzoyl) Peroxide (**4**)

Demineralized water (25 mL) was measured into a round-bottomed flask and cooled to 0–5 °C. Sodium peroxide (3.0 g, 0.04 mol) was added. A solution of 4-trifluoromethoxybenzoyl chloride (11.2 g, 0.05 mol) in toluene (25 mL) was then added immediately. The reaction flask was stoppered and the mixture stirred vigorously for 2 h. The peroxide product was filtered off, washed with cold demineralised water and dried. It was obtained as a white powder (5.95 g, 58% yield). After recrystallization from dry toluene (100 mL), it had:

Mpt: 73–75 °C.

IR Spectrum: ν_max_ 3113 (Ar C–H), 1766 (C=O), 1606 (C=C), 1505 (C–C), 1279 (C–O), 1208 cm^−1^

(C–F).

^1^H-NMR Spectrum (in CD_2_Cl_2_): δ 7.45 (d, 4H), 8.2 ppm (d, 4H).

^19^F-NMR Spectrum (in CD_2_Cl_2_): δ -53 ppm.

#### Appendix A.2.2. Synthesis of CTA (**5**)

*(a) 2-(4-Trifluoromethyl)phenylpropene*: A sodium hydride dispersion in mineral oil (60% by weight sodium hydride, 1.6 g, 0.04 mol) was added to a solution of methyltriphenylphosphonim bromide (12.9 g, 0.036 mol) in anhydrous diethyl ether (50 mL) in a round-bottomed flask. The mixture was stirred under reflux (~45 °C), under dry nitrogen, for 3 h. 4-Trifluoromethylacetophenone (4.7 g, 0.025 mol) in anhydrous diethyl ether (15 mL) was added dropwise and the reaction mixture was stirred under reflux (~45 °C) overnight (16 h). The reaction mixture was added to demineralized water (100 mL) and the product was extracted with dichloromethane (3×, 50 mL). The combined dichloromethane extracts were dried and concentrated and the product mixture was dried. The desired product was distilled under reduced pressure from the crude reaction product in a Kugelröhr apparatus (Büchi UK, Newmarket, UK). The product was an oil (3.07 g, 66% yield). It had:

Bpt: 174–174.5^o^C

MS (CI) m/z: 186 (M, 100%), 117 (37%), 59 (42%).

IR Spectrum: ν_max_ 3088 (Ar C–H), 3050 (Ar C–H), 2975 (C–H), 2946 (C–H), 1631 (C=C), 1600

(C=C), 1495 (C–C), 1442 (C–C), 1233 cm^−1^ (C–F).

^1^H-NMR Spectrum: δ 2.2 (s, 3H), 5.15 (d, 1H), 5.42 (d, 1H), 7.25–7.6 ppm (m, 4H).

^19^F-NMR Spectrum: δ -59 ppm (s).

*(b) 4-Fluorodithiobenzoic acid*: Magnesium turnings (2.0 g, 0.082 mol), a catalytic amount of iodine, 1-bromo-4-fluorobenzene (1.0 g) and dry tetrahydrofuran (10 mL) were placed in a dry round-bottomed flask in an ice-water bath. The flask was flushed with dry nitrogen for 20 min. When the Grignard reaction commenced, a solution of 1-bromo-4-fluorobenzene (14 g, 0.08 mol) in dry tetrahydrofuran (30 mL) was added dropwise over 30 min. The Grignard reaction finished in 1.5 h. Carbon disulphide (6.1 g, 0.08 mol) was then added dropwise over 20 min, maintaining the reaction temperature below 20 °C. The reaction mixture was stirred at 20 °C under dry nitrogen for a further 1 h. The product was hydrolyzed by the slow addition of cold distilled water (100 mL). The salts formed, along with any excess magnesium turnings, were removed by filtration. The tetrahydrofuran solvent was removed under reduced pressure. The remaining reaction mixture was acidified to pH 1 by dropwise addition of fuming hydrochloric acid (37%). The colour of the reaction solution changed from brown-red to a permanent deep purple. The product was extracted with diethyl ether (3×, 200 mL). The organic phase was dried and concentrated under reduced pressure. The product was a purple oil (10.37 g, 84% yield). This was used immediately to prepare CTA (**5**).

*(c) CTA (**5**):* 4-Fluorodithiobenzoic acid (10.34 g, 0.07 mol) was dissolved in carbon tetrachloride (80 mL), in a round-bottomed flask. 2-(4-Trifluoromethylphenyl)propene (13.96 g, 0.075 mol) was added dropwise over 15 min. The reaction mixture was stirred under reflux (~70 °C), under dry nitrogen, for 18 h. The carbon tetrachloride solvent was then removed under reduced pressure, yielding the crude reaction product (19.12 g). The crude product was subjected to flash column chromatography on aluminium oxide (Aldrich, activated, neutral, Brockmann 1, grade 2, ca. 150 mesh), using hexane as the eluent. This gave the desired pure product as a viscous purple oil (7.34 g, 34% yield). It had:

MS (CI) m/z: 186 (100%), 117 (78%), 49 (71%).

EA: Expected%: C 55.48, H 4.07, S 18.51, F 21.97; Found%: C 55.23, H 3.82, S 18.56, F 22.39.

IR Spectrum: ν_max_ 3067 (Ar C–H), 2970 (C–H), 2928 (C–H), 2869 (C–H), 1617 (C–C), 1594 (C–C),

1498 (C–C), 1409 (C–C), 1328 (Ar C–F), 1235 cm^−1^ (C–F).

^1^H-NMR Spectrum: δ 2.0 (s, 6H), 7.0–8.0 ppm (m, 8H).

^19^F-NMR Spectrum: δ -59 (s, CF_3_), −104 ppm (m, ArF).

#### Appendix A.2.3. Synthesis of E-4,4′-Bis(Trifluoromethyl)stilbene (**6**)

*(a) 4-Trifluoromethylbenzyltriphenylphosphonium chloride:* 4-Trifluoromethylbenzyl chloride (15.2 g, 0.08 mol) and triphenylphosphine (23.0 g, 0.088 mol) were weighed into a round-bottomed flask. Chloroform was added to dissolve the reactants. The reaction solution was stirred under reflux (~65 °C), under dry nitrogen for 48 h. The reaction solution was then concentrated under reduced pressure. A large excess of cold diethyl ether was added to dissolve any reactants and precipitate the salt product. The precipitate was filtered off and dried. The product was a white solid (36.2 g, 100% yield). It had:

Mpt: 348–350 °C.

EA: Expected%: C 68.35, H 4.63, Cl 7.76, P 6.78, F 12.48; Found%: C 68.10, H 4.23, Cl 8.99, P 6.08,

F 12.60.

IR Spectrum: ν_max_ 3041 (Ar C–H), 2856 (C–H), 2785 (C–H), 1586 (C=C), 1435 (C–C), 1110 cm^−1^

(C–F).

^1^H-NMR Spectrum: δ 5.9–6.1 (d, 2H), 7.2–7.9 ppm (m, 19H).

^19^F-NMR Spectrum: δ -59 ppm (s).

*(b) E-4,4′-Bis-(trifluoromethyl)stilbene (**6**):* 4-Trifluoromethylbenzyltriphenyl phosphonium chloride (23.76 g, 0.052 mol) and 4-trifluoromethylbenzaldehyde (5.97 g, 0.034 mol) were weighed into a round-bottomed flask. Tetra-n-butylammonium bromide (phase transfer catalyst, 0.83 g, 0.003 mol), 10% aqueous sodium hydroxide (80 mL) and dichloromethane (80 mL) were added. The reaction mixture was stirred vigorously under dry nitrogen for 96 h. The product was extracted with dichloromethane (3×, 50 mL). The combined extracts were washed with demineralized water (5×, 100 mL), dried and concentrated under reduced pressure. TLC analysis, using hexane as the eluent, revealed a mixture of species present. Therefore, the product was passed through a column of aluminium oxide (Aldrich, activated, neutral, Brockmann 1, grade 1, ca. 150 mesh), using hexane as the eluent. Product containing fractions were combined and concentrated under reduced pressure and the products were dried. The *Z*-isomer was an oil and the *E*-isomer (**4**) a white solid (total mass 8.06 g, 75%; ratio of *Z:E* isomers 2.3:1.0). The *E*-isomer had:

Mpt: 130–131 °C, cf. literature value of 124–127 °C [22].

IR Spectrum: ν_max_ 3084 (Ar C–H), 3051 (Ar C–H), 1614 (C=C), 1418 (C–C), 1328 cm^−1^ (C–F).

^1^H-NMR Spectrum: δ 7.2–7.8 ppm (m, 10H).

^19^F-NMR Spectrum: δ -59 ppm (s).

#### Appendix A.2.4. Synthesis of 1,1-Bis-(4-Trifluoromethylphenyl)ethene (**7**)

*(a) 1,1-bis-(4-trifluoromethylphenyl)ethanol:* Magnesium turnings (3.16 g, 0.13 mol), a catalytic quantity of iodine and dry tetrahydrofuran (10 mL) were placed in a dry round-bottomed flask and flushed with dry nitrogen for 20 min. 4-Trifluoromethylbromobenzene (28.6 g, 0.127 mol) in dry tetrahydrofuran (60 mL) was added dropwise. Mild heating was used to commence the reaction. The remaining 4-trifluoromethylbromobenzene solution was added at a rate to maintain a steady reflux. The mixture was stirred at room temperature, under dry nitrogen, for 2 h. The reaction solution was cooled in ice to 0–5 °C. Ethyl acetate (2.25 g, 0.255 mol) dissolved in dry tetrahydrofuran (30 mL) was added dropwise over 15 min and the solution was heated under reflux (~75 °C) for 3 h. The reaction solution was cooled to 0–5 °C. Saturated aqueous sodium carbonate solution (50 mL) and 5% sodium thiosulphate solution (50 mL) were added. The product was extracted with diethyl ether (3×, 100 mL). The combined organic extracts were washed with demineralised water (100 mL), dried and concentrated under reduced pressure. The dried crude product, 1,1-bis-(para-trifluoromethylphenyl)ethanol, was a brown crystalline solid (17 g, 98% yield) and was used in further reactions without purification. It had:

Bpt: 334–336 °C, c.f. literature value of 330 °C [23].

^1^H-NMR Spectrum: δ 2.1 (s, 3H), 7.15–7.4 ppm (dd, 8H).

*(b) 1,1-Bis-(4-trifluoromethylphenyl)ethene (**7**):* 1,1-Bis-(*p*-trifluoromethylphenyl)ethanol, potassium hydrogen sulphate (4.1 g, 0.03 mol) and chloroform (20 mL) were placed in a round-bottomed flask and stirred under reflux (~65 °C) for 3 h. The mixture was cooled and filtered. The filtrate was added to demineralized water (50 mL). The product was extracted with dichloromethane (3×, 50 mL). The combined organic extracts were dried and concentrated under reduced pressure. The dried product was an oil (4.65 g, 58% yield) which slowly crystallized. It had:

Mpt: 31–33 °C, cf. literature value of 33–34 °C [24].

IR spectrum: ν_max_ 3096 (Ar C–H), 3052 (Ar C–H), 1690 (C=C), 1616 (C=C), 1409 (C–C), 1328 cm^−1^

(C–F).

^1^H-NMR Spectrum: δ 5.65 (s, 2H), 7.4–7.8 ppm (m, 8H).

^19^F-NMR Spectrum: δ - 59 ppm (s).

#### Appendix A.2.5. 1,1-Bis-(4-(4,4,4-Trifluorobutoxy)phenyl)ethane (**8**)

*(a) 4,4′-di(4,4,4-trifluorobutoxy)benzophenone:* Potassium iodide (0.17 g, 0.001 mol), potassium carbonate (3.45 g, 0.05 mol) and anhydrous N,N-dimethylformamide (25 mL) were measured into a dry round-bottomed flask, which was cooled in an ice-water bath and flushed with dry nitrogen for 15 min. Then, 4,4′-Dihydroxybenzophenone (1.93 g, 0.009 mol), in anhydrous N,N-dimethylformamide (25 mL), was added dropwise over 15 min. The reaction solution was stirred under reflux (~130 °C), under dry nitrogen, for 3 h. The reaction mixture was then cooled in an ice-water bath and 1-bromo-4,4,4-trifluorobutane (4.0 g, 0.021 mol) in anhydrous N,N-dimethylformamide (25 mL) was added dropwise over 15 min. The reaction mixture was stirred under reflux (~90 °C), under dry nitrogen, for 17 h. Once cooled, the reaction solution was poured into 10% hydrochloric acid (100 mL). The desired reaction products were extracted using dichloromethane (3×, 100 mL) and the combined extracts were washed with demineralized water (10×, 100 mL), dried and concentrated under reduced pressure. The product was recrystallized from hot absolute ethanol. It was obtained as a white-pale yellow crystalline solid (3.86 g, 99% yield). It had:

Mpt: 163–165 °C.

EA: Expected%: C 58.07, H 4.64, F 26.24; Found%: C 57.59, H 4.70, F 26.19.

IR Spectrum: ν_max_ 2955 (Ar C–H), 2886 (C–H), 1642 (C=O), 1600 (C=C), 1480 (C–C), 1253 (C–F),

1151 cm^−1^ (C–O).

^1^H-NMR Spectrum: δ 2.05–2.2 (m, 4H), 2.3–2.5 (m, 4H), 4.1–4.2 (t, 4H), 6.95–7.05 (d, 2H), 7.75–7.9

ppm (d, 2H).

^19^F-NMR Spectrum: δ -58 ppm (m).

*(b) 1,1-Bis-(4-(4,4,4-trifluorobutoxy)phenyl)ethene (**8**)*: A sodium hydride dispersion in mineral oil (60% by weight sodium hydride, 0.8 g, 0.02 mol) was added to a suspension of methyltriphenylphosphonium bromide (6.43 g, 0.018 mol) in anhydrous diethyl ether (25 mL) in a round-bottomed flask. The mixture was stirred and heated under reflux (~50 °C) under dry nitrogen for 4 h. The reaction mixture was cooled and a solution of 4,4′-bis-(4,4,4-trifluoro- butoxy)benzophenone (3.8 g, 0.009 mol), in anhydrous diethyl ether (10 mL), was added dropwise over 30 min. The solution was stirred under reflux (~50 °C), under dry nitrogen, for 17 h. The reaction mixture was then added to demineralized water (100 mL) and the products were extracted with dichlormethane (3×, 100 mL). The combined extracts were dried and concentrated under reduced pressure. TLC analysis, using dichloromethane as the eluent, revealed a mixture of species to be present. Hence, the product mixture was subjected to column chromatography on silica gel using dichloromethane as eluent. Product-containing fractions were combined and concentrated under reduced pressure and dried. Removal of the solvent under vacuum left the product as a pale yellow crystalline solid (3.71 g, 98% yield). It had:

Mpt: 77–79 °C

EA: Expected%: C 61.11, H 5.13, F 26.36; Found%: C 61.14, H 5.91, F 25.74.

IR Spectrum: ν_max_ 2954 (Ar C-H), 2925 (Ar C–H), 2871 (C–H), 1608 (C=C), 1475 (C–C), 1453 (C–

C), 1245 (C–F), 1152 cm^−1^ (C–O).

^1^H-NMR Spectrum: δ 2.05–2.2 (m, 4H), 2.3–2.5 (m, 4H), 4.05–4.15 (t, 4H), 5.3–5.4 (s, 2H), 6.85–

6.95 (d, 4H), 7.25–7.35 ppm (d, 4H).

^19^F-NMR Spectrum: δ -58 ppm (m).

#### Appendix A.2.6. Methyl E-3-Trifluoromethylcinnamate (**9**)

Pyridine (6.69 g, 0.084 mol), methanol (3.17 g, 0.098 mol) and dichloromethane (50 mL) were placed in a round-bottomed flask, which was cooled in an ice-water bath. E-3-trifluoromethylcinnamoyl chloride (13.84 g, 0.059 mol), dissolved in dichloromethane (50 mL), was added dropwise over 20 min. The reaction mixture was brought to room temperature and stirred under dry nitrogen for 67 h. The mixture was then washed with demineralized water (4×, 50 mL), 2 M hydrochloric acid (100 mL), saturated sodium hydrogen carbonate solution (100 mL) and saturated sodium chloride solution (100 mL). The dichloromethane solution was dried and concentrated under reduced pressure. The product was dried. The product, methyl E-3-trifluoromethylcinnamate (9), was obtained as a white crystalline solid (7.55 g, 56% yield). It had:

Mpt: 34–36 °C.

EA: Expected%: C 57.40, H 3.90, F 24.80; Found%: C 57.43, H 4.01, F 24.62.

IR Spectrum: ν_max_ 3081 (Ar C–H), 2954 (C–H), 1723 (C=O), 1643 (C=C), 1442 (C–C), 1336 (C–F),

1166 cm^−1^ (C–O).

EA: Expected%: C 57.40, H 3.90, O 13.90, F 24.80; Found%: C 57.43, H 4.01, F 24.62.

^1^H-NMR Spectrum: δ 3.85 (s, 3H), 6.5–6.65 (d, 1H), 7.5–7.9 ppm (m, 5H).

^19^F-NMR Spectrum: δ -59 ppm (s).

#### Appendix A.2.7. Synthesis of 4,4,4-Trifluorobutyl E-Cinnamates (**10**)–(**12**)

*(a) Synthesis of 4,4,4-Trifluorobutyl E-Cinnamate (**10**)*: Pyridine (0.55 g, 0.007 mol), 4,4,4-trifluorobutan-1-ol (1 g, 0.008 mol) and anhydrous dichloromethane (10 mL) were measured into a round-bottomed flask. The solution was stirred under dry nitrogen in an ice-water bath for 20 min. *E*-Cinnamoyl chloride (0.43 g, 0.003 mol) in anhydrous dichloromethane (10 mL) was added dropwise over 10 min. The reaction solution was allowed to come to room temperature and was then stirred for 90 h under dry nitrogen. The reaction mixture was washed with demineralized water (4×, 25 mL), 2 M hydrochloric acid (50 mL), saturated aqueous sodium hydrogen carbonate solution (50 mL) and saturated brine (50 mL). The dichloromethane extract was dried and concentrated under reduced pressure. TLC analysis, using 20% diethyl ether in 40–65 °C petroleum ether as the eluent, revealed a mixture of species to be present. Hence, the product mixture was subjected to column chromatography on silica gel using 20% diethyl ether in 40–65 °C petroleum ether as the eluent. Product containing fractions were combined and concentrated under reduced pressure and the product was dried. The product was a pale yellow oil (0.27 g, 40% yield). It had:

Bpt: 288–292 °C.

EA: Expected%: C 60.46, H 5.07, F 22.07; Found%: C 60.29, H 5.30, F 21.95.

IR Spectrum: ν_max_ 3063 (Ar C–H), 3030 (Ar C–H), 2961 (C–H), 2901 (C–H), 1723 (C=O), 1639

(C=C), 1451 (C–C), 1177 (C–F), 1051 cm^−1^ (C–O).

^1^H-NMR Spectrum: δ 1.8–2 (p, 2H), 2.1–2.3 (m, 2H), 4.15–4.3 (t, 2H), 6.35–6.45 (d, 1H), 7.3–7.4 (m,

3H), 7.4–7.55 (m, 2H), 7.6–7.75 ppm (d, 1H).

^19^F-NMR Spectrum: δ -58 ppm (m).

*(b) Synthesis of 4,4,4-Trifluorobutyl E-4-Methoxycinnamate (**11**):* 4,4,4-Trifluorobutyl *E*-p-methoxycinnamate (**11**) was synthesized using a similar method to above but starting with *E*-3-methoxycinnamoyl chloride (0.79 g, 0.004 mol). The reaction was carried out for 22 h. The product mixture was treated to column chromatography on silica gel using 50:50 diethyl ether: 40–65 °C petroleum ether as the eluent. The product was a pale yellow oil (0.53 g, 46% yield). It had:

Bpt: 268–271 °C.

EA: Expected%: C 58.33, H 5.25, F 19.77; Found%: C 59.30, H 5.65, F 18.93.

IR Spectrum: ν_max_ 3064 (Ar C–H), 2961 (C–H), 2838 (C–H), 1713 (C=O), 1640 (C=C), 1454 (C–C),

1435 (C–C), 1158 (C–F), 1048 cm^−1^ (C–O).

^1^H-NMR Spectrum: δ 1.95–2.1 (p, 2H), 2.2–2.4 (m, 2H), 3.85–3.95 (s, 3H), 4.25–4.35 (t, 2H), 6.4–

6.55 (d, 1H), 6.95–7.05 (d, 1H), 7.05–7.15 (s, 1H), 7.15–7.2 (d, 1H), 7.3–7.4 (q, 1H), 7.65–7.8 ppm

(d, 1H).

^19^F-NMR Spectrum: δ -58 ppm (m).

*(c) Synthesis of 4,4,4-Trifluorobutyl E-3-Methoxycinnamate (**12**)*: 4,4,4-Trifluorobutyl *E*-p-methoxycinnamate was synthesized using the same method but starting with *E*-4-methoxycinnamoyl chloride (0.79 g, 0.004 mol). The product mixture was subjected to column chromatography on silica gel using 40% diethyl ether in 40–65 °C petroleum ether as the eluent. The product was a pale yellow-green oil (0.4 g, 35% yield). It had:

Bpt: 280–283 °C.

EA: Expected%: C 58.33, H 5.25, F 19.77; Found%: C 58.78, H 5.47, F 19.22.

IR Spectrum: ν_max_ 3059 (Ar C–H), 2961 (C–H), 2905 (C–H), 2841 (C–H), 1712 (C=O), 1634 (C=C),

1464 (C–C), 1455 (C–C), 1164 (C–F), 1026 cm^−1^ (C–O).

^1^H-NMR Spectrum: δ 1.95–2.1 (p, 2H), 2.2–2.4 (m, 2H), 3.85–3.95 (s, 3H), 4.25–4.35 (t, 2H), 6.3–6.4

(d, 1H), 6.95–7 (d, 2H), 7.45–7.6 (d, 2H), 7.6–7.8 ppm (d, 1H).

^19^F-NMR Spectrum: δ -58 ppm (m).
